# Post-Hemorrhagic Hydrocephalus and Outcomes Amongst Neonates With Intraventricular Hemorrhage: A Systematic Review and Pooled Analysis

**DOI:** 10.7759/cureus.18877

**Published:** 2021-10-18

**Authors:** Candida Pinto, Preeti Malik, Rutikbhai Desai, Vrushali Shelar, Daria Bekina-Sreenivasan, Travis A Satnarine, Liseth K Lavado, Ramit Singla, Devraj Chavda, Surabhi Kaul, Shae Datta, Shamik Shah, Urvish K Patel

**Affiliations:** 1 Public Health, Icahn School of Medicine at Mount Sinai, New York, USA; 2 Pathology, Montefiore Medical Center, Wakefield Campus, Bronx, USA; 3 Internal Medicine, University of North Carolina Cardiology at Nash, Rocky Mount, USA; 4 Internal Medicine, Saratov State Medical University, Saratov, RUS; 5 Pediatric Medicine, Tyumen State Medical University, Tyumen, RUS; 6 Neonatal Intensive Care Unit, Port of Spain General Hospital, Port of Spain, TTO; 7 Nursing, Rutgers School of Nursing, Newark, USA; 8 Pediatric Neurology, Children's Hospital of Michigan, Detroit, USA; 9 Pediatric Neurology, State University of New York Downstate Medical Center, Brooklyn, USA; 10 Pediatric Neurology, Mercyone North Iowa, Mason City, USA; 11 Neurology, NYU Langone Health, New York, USA; 12 Neurology, Stormont Vail Health, Topeka, USA; 13 Neurology and Public Health, Icahn School of Medicine at Mount Sinai, New York, USA

**Keywords:** intraventricular hemorrhage, neonates, newborn, hydrocephalus, preterm

## Abstract

Introduction

Intraventricular hemorrhage (IVH) is a common cause of morbidity and mortality in preterm neonates. IVH leads to complications such as posthemorrhagic hydrocephalus (PHH), which commonly occurs in neonates with a more severe degree of IVH. Hence, we aimed to evaluate the characteristics and outcomes of PHH in neonates with IVH.

Methods

We performed a systematic review of cases reported from January 1978 to December 2020 through the PubMed database, using Preferred Reporting Items for Systematic Reviews and Meta-Analyses (PRISMA) guidelines and the keywords ‘intraventricular hemorrhage,’ ‘cerebral intraventricular hemorrhage,’ and ‘newborn.’ A total of 79 articles were considered for analysis, and data on neonatal and maternal characteristics and outcomes were collected. The analysis was performed by using the χ2 test, Wilcoxon rank-sum test, and multivariate logistic regression model.

Results

We analyzed a total of 101 IVH cases, 54.5% were male and 62.4% preterm. Thirteen point nine percent (13.9%) presented with grade I, 35.6% grade II, and grade III respectively, and 8% grade IV IVH. Among the 59 (58.4%) neonates with PHH, 33.6% had resolved PHH and 24.8% had unresolved. In adjusted regression analysis, we found that neonates with resolved PHH have lower odds of having neurodevelopmental delay (OR:0.15, 95%CI:0.03-0.74; p=0.02) and death (OR:0.9;95%CI:0.01-0.99; p=0.049) as compared to unresolved PHH.

Conclusion

Our study showed that neonates with resolved PHH have a statistically significant lower risk of neurodevelopmental delay (NDD) and mortality. Future studies should be planned to evaluate the role of treatment and its effect on outcomes in IVH neonates with PHH as a complication.

## Introduction

Intraventricular hemorrhage (IVH) remains a significant cause of morbidity and mortality in newborns, particularly in preterm infants [1]. IVH in preterm infants occurs due to the rupture of fragile blood vessels within the germinal matrix of the developing brain into the ventricular system and fluctuations in the cerebral blood flow. IVH is described in four grades: (1) Bleeding in a small area of the ventricles, (2) Bleeding inside the ventricles, (3) Ventricles are enlarged by the blood, and (4) Bleeding into the brain tissues around the ventricles, of which grade IV is the most severe form of bleeding [2]. Despite the considerable prevalence of IVH in preterm babies, this phenomenon can also occur in term newborns and is accompanied by different etiologies, clinical manifestations, and outcomes [3]. Around 90% of cases of IVH occur within the first three days of life and 20-40% extend during the first week of life. Approximately 60% of premature infants with grade III and IV IVH will incur neurocognitive problems such as motor dysfunction, coordination problems, cerebral palsy, language and learning disabilities, attention deficit-hyperactivity disorder, behavioral issues, and social-emotional difficulties.

IVH leads to complications such as posthemorrhagic ventricular dilatation (PHVD), with or without post-hemorrhagic hydrocephalus (PHH), and periventricular hemorrhagic infarction (PHI) [1]. PHH is a serious complication that occurs in up to 35% of preterm neonates with IVH [4]. It occurs more frequently in neonates with more severe degrees of IVH and can lead to long-term neurological impairment and increased mortality. Treatment modalities for PHH include serial lumbar punctures, ventricular taps, insertion of external ventricular drainage, or placement of ventriculoperitoneal (VP) shunt [4]. Neonates with VP shunt are prone to develop shunt infections, malfunctions, and have worse neurodevelopmental outcomes [5]. Despite many treatment options, there is still no consensus regarding the management of PHH. There is limited literature of case reports and case series on the outcomes of PHH in neonates with IVH. Hence, we sought to perform a systematic review to evaluate the epidemiologic characteristics of neonates with IVH, with a primary focus on the characteristics and outcomes of PHH among neonates with IVH.

## Materials and methods

We performed a systematic review of IVH cases reported in both preterm and term neonates and followed the predesigned Preferred Reporting Items for Systematic Reviews and Meta-Analyses (PRISMA) protocol and standard for reporting the systematic review to the best extent of our possibilities [6].

Aims

The primary aim of the study was to compare the outcomes, including neurodevelopmental delay and death, in neonates with resolved PHH vs. unresolved PHH. The secondary aim was to evaluate the epidemiological characteristics of neonates with IVH and PHH.

The neurodevelopmental delay includes all patients with neurological impairment like gross and fine motor skills, language, and cognitive skills.

Search strategy and eligibility criteria

A comprehensive search was conducted using the PubMed database. We included case reports, case illustrations, letters reporting human cases, and case series from January 1978 to December 2020 by using the keywords ‘intraventricular hemorrhage,’ ‘cerebral intraventricular hemorrhage,’ ‘newborn,’ ‘neonate.’ Manual checks of the reference list were performed. Any articles that were written in the English language and describing details of IVH in term or preterm neonates were included. After the initial search, all titles and abstracts were reviewed. Finally, each article that met the criteria underwent a full-text review. Animal studies, non-English studies, and non-full text studies were excluded.

Selection of studies and data collection

**Figure 1:**
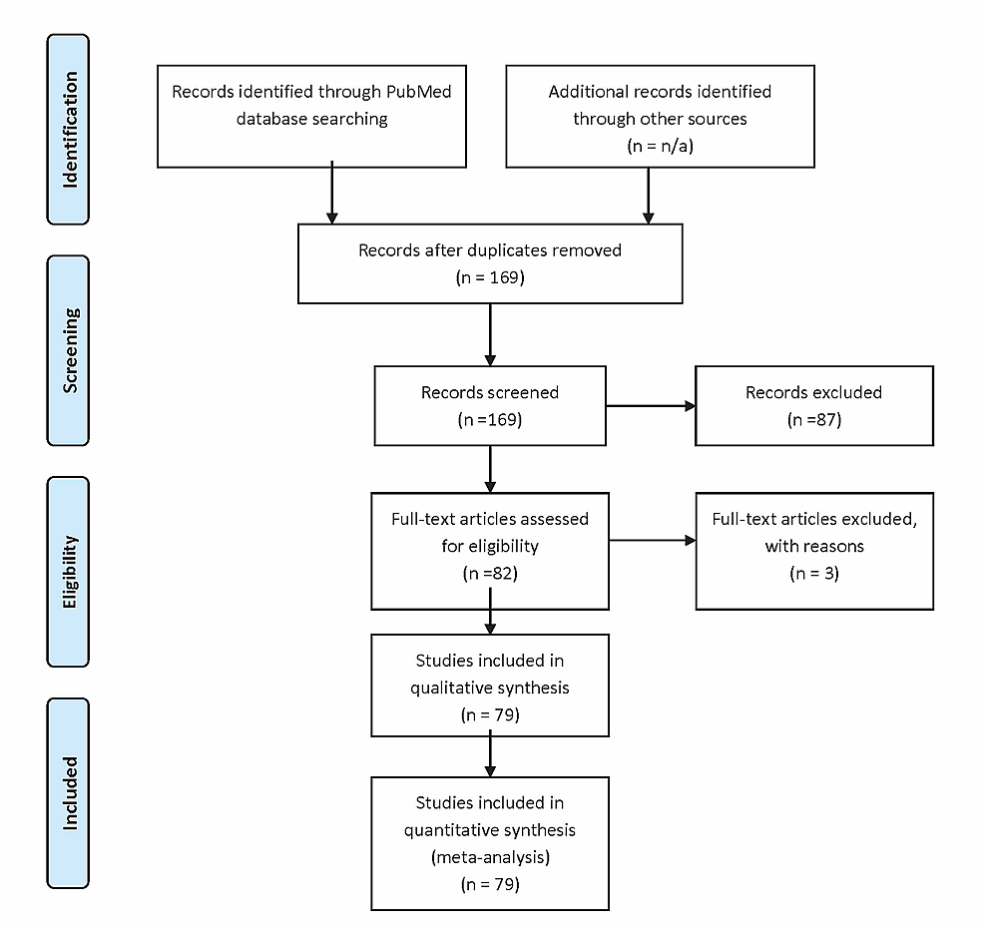
Preferred Reporting Items for Systematic Reviews and Meta-Analyses (PRISMA) flow diagram of the literature search and selection process of intraventricular hemorrhage in neonates

By using this search strategy, a total of 169 articles were identified and screened. After initial screening, 87 articles were excluded, as the onset of IVH was more than 28 days after birth, there was incomplete information on demographics or IVH onset, or they were not well-defined or were difficult to comprehend. Two physicians (CP and PM) independently reviewed all 82 articles, and they were further verified by a third independent reviewer (UP). Any conflict was resolved by consensus. Three articles were excluded, as they did not contain information regarding gestational age. This left us with 79 case reports and case series consisting of 101 cases that were considered for qualitative and quantitative analysis. Figure 1 depicts a flow chart of the literature search and selection process, and Table 1 outlines the studies considered for analysis with age and gender.

**Table 1:** Distribution of 101 intraventricular hemorrhage cases based on age and gender

Author; Year	Number of Cases	Gestational Age (weeks)	Gender
Akisu et al.; 2003 [7]	3	38	M
		30	F
		28	M
Alhifany et al.;2017 [8]	1	24	F
Alvarado & Rodriguez; 2017 [9]	1	27	Not given
Arai et al.; 2018 [10]	1	30	M
Arvanitis et al.; 1996 [11]	1	36	F
Benvenisti et al.; 2015 [12]	1	38	F
Bhattacharya et al.; 2018 [13]	1	37	M
Biermayr et al.; 2016 [14]	1	30	F
Bilguvar et al.; 2009 [15]	2	24	F
		24	M
Borenstein-Levin et al.; 2014 [16]	2	29	M
		24	M
Brown et al.; 1994 [17]	2	28	M
		26	M
Castro Conde et al.; 2005 [18]	1	37	M
Cheng et al.; 2006 [19]	1	38	M
Chun et al.; 2011 [20]	1	26	F
Counsell et al.; 1999 [21]	1	28	F
Duijvestijn et al.; 2003 [22]	1	40	M
Eller & Pasternak; 1985 [23]	4	31	M
		27	M
		32	M
		30	M
Fawer & Levene; 1982 [24]	2	30	M
		30	M
Fawer & Dubowitz; 1983 [25]	1	30	M
Friese et al.; 2003 [26]	1	42	M
Fusch et al.; 1997 [27]	1	36	Not given
Golinko et al.; 2018 [28]	1	23	Not given
Hanigan et al.; 1990 [29]	1	39	F
Hashimoto et al.; 1997 [30]	1	36	M
Heafner et al.; 1985 [31]	1	38	F
Heck et al.; 20021 [32]	1	36	F
Heineiking et al.; 2003 [33]	1	39	M
Hentschel et al.; 1993 [34]	1	28	F
Hevner et al.; 1997 [35]	1	28	M
Hill et al.; 1984 [36]	1	38	Not given
Jacob et al.; 2017 [37]	1	35	F
Kamikawa et al.; 2001 [38]	2	36	F
		29	M
Katumba et al.; 2005 [39]	1	25	Not given
Khatri et al.; 2018 [40]	1	38	F
Kimura et al.; 2018 [41]	1	24	M
Ko et al.; 2010 [42]	1	30	Not given
Koehne et al.; 2006 [43]	1	27	M
Krueger et al.; 2008 [44]	1	28	Not given
Kumar et al.; 2013 [45]	1	41	F
Mathews et al.; 2017 [46]	1	38	M
Mays et al.; 1995 [47]	3	24	M
		27	F
		24	F
Ment et al.; 1984 [48]	2	26	F
		28	M
Mohila et al.; 2010 [49]	2	33	F
		33	F
Nakano et al.; 2007 [50]	1	24	F
Negishi et al.; 1989 [51]	1	38	M
Nelson et al.; 1986 [52]	1	36	M
Pasternak & Volpe; 1979 [53]	1	35	F
Prats et al.; 2001 [54]	1	29	F
Preub et al.; 2015 [55]	1	29	M
Ramenghi et al.; 2002 [56]	1	33	M
Ritschl et al.; 1987 [57]	1	30	M
Scher et al.; 1982 [58]	5	39	F
		42	M
		39	M
		40	M
		40	F
Seeburg et al.; 2014 [59]	2	24	Not given
		37	Not given
Shackleford et al.; 1984 [60]	1	28	Not given
Sharma et al.; 2012 [61]	1	28	M
Shirane et al.; 1986 [62]	1	38	F
Sobolewska et al.; 2018 [63]	1	41	M
Suksumek et al.; 2013 [64]	1	39	M
Szpecht et al.; 2016 [65]	2	39	M
		39	M
Tajdar et al.; 2018 [66]	1	34	M
Tan et al.; 1998 [67]	1	38	M
Van Raay et al.; 2009 [68]	1	38	F
Voutsinas et al.; 1991 [69]	1	38	M
Weinschenk et al.; 2001 [70]	1	27	M
Whitelaw et al.; 1984 [71]	1	37	F
Mitchell et al.; 1980 [72]	1	38	F
Timothy et al.; 1979 [73]	1	41	F
Wehberg et al.; 1991 [74]	1	38	M
Ma et al.; 2017 [75]	1	38	M
Abel et al.; 2003 [76]	1	38	F
De Vries et al.; 2000 [77]	3	28	F
		27	M
		40	M
Filippi et al; 2004 [78]	1	25	F
Ho et al.; 1987 [79]	1	30	M
Molnar et al.; 2012 [80]	1	28	M
Shah et al.; 2010 [81]	1	24	Not given
Tancabelic et al.; 2004 [82]	1	36	M
Yang et al.; 1999 [83]	1	38	F
Yu et al.; 1994 [84]	1	26	M
Upma et al.; 2016 [85]	1	38	M

All eligible studies were reviewed using a standardized web-based form to collect information. All data were summarized, which included neonatal characteristics such as gestational age, birth weight, sex, IVH onset, IVH grade, associated complications like seizures, respiratory distress syndrome, cardiovascular or neurological diseases, presence of PHH, resolved or treated by serial lumbar puncture or permanent ventriculoperitoneal shunt, and maternal characteristics such as maternal age, mode of delivery and maternal steroid use. Data on outcomes of neurodevelopmental delay and mortality were collected.

Statistical analysis

A Microsoft Excel sheet (Microsoft Corporation, Redmond, WA) was used to collect the data of 101 patients, and the data were analyzed using SAS 9.4 (SAS Institute Inc., Cary, NC). We calculated frequency, percentage, and median and standard error for the characteristics of the patients with IVH and PHH. Univariate analysis of categorical data was performed using the chi-square test and differences in the median were evaluated by the Wilcoxon-Mann-Whitney test. Multivariable logistic regression analysis was performed to evaluate the association between outcomes and PHH. Odds ratio, 95% confidence interval, and goodness of fit (c-value) were calculated, and p<0.05 was considered significant. Models were adjusted with gestational age (term vs. preterm), sex, IVH onset, and IVH laterality. We have not adjusted models with maternal characteristics due to a lack of evidence from previous research to establish a relationship between neonatal mortality and neurodevelopmental delay (NDD) with post-PHH resolution. Due to insufficient data on the method of resolution (without intervention, serial lumbar puncture, and VP shunt), we have not included them in the regression analysis.

## Results

Neonatal and maternal characteristics of IVH

We included a total of 101 individual cases of IVH in newborns, of which 17 (16.8%) had unilateral and 65 (64.4%) had bilateral IVH. Fifty-five (54.5%) were male and 35 (34.6%) female. Of the 101 neonates, 14 (13.9%) presented with grade I, 36 (35.6%) with grade II, 36 (35.6%) grade III, and eight (8%) grade with grade IV IVH. Imaging studies, such as head ultrasound, were performed in 69 (68.3%), MRI brain in 20 (20%), and CT scan in 37 (36.6%). Fifty-nine (58.4%) neonates developed PHH, which was resolved in 34 (33.6%) and unresolved in 25 (24.8%). Twenty-three (22.7%) underwent serial lumbar puncture (LP) and a permanent VP shunt was placed in 37 (36.6%). NDD occurred in 34 (33.7%) and 17 (16.8%) died from complications of IVH (Table 2).

**Table 2:** Maternal and clinical characteristics of neonates with intraventricular hemorrhage (IVH) *IVH Grade: 1-Bleeding is confined to the germinal matrix; 2-Germinal matrix hemorrhagic and IVH occupies <50% of the lateral ventricle volume; 3-Germinal matrix hemorrhagic and IVH occupies more than 50% of the lateral ventricle volume and is associated with acute ventricular distension; 4-Periventricular hemorrhagic infarction; Hemorrhagic infarction in periventricular white matter ipsilateral to large IVH. # Neurodevelopmental delay includes all patients with neurological impairment like gross and fine motor skills, language, and cognitive skills.

Variables	Intraventricular Hemorrhage N=101 (%)
Intraventricular Hemorrhage (IVH)	
Unilateral	17 (16.8%)
Bilateral	65 (64.4%)
Laterality Unknown	19 (18.8%)
Post Hemorrhagic Hydrocephalus (PHH)	
Yes	59 (58.4%)
No	42 (41.6%)
PHH Resolved	
Yes	34 (33.6%)
No	25 (24.8%)
Neonatal Characteristics	
Birth Weight (Grams)	
Median (IQR)	1728 (2189)
Gender	
Male	55 (54.5%)
Female	35 (34.6%)
Gestational Age	
Preterm	63 (62.4%)
Term	38 (37.6%)
Ultrasound	
Yes	69 (68.3%)
No	32 (31.7%)
Magnetic Resonance Imaging (MRI)	
Yes	20 (20%)
No	81 (80%)
Computed Tomography (CT) Scan	
Yes	37 (36.6%)
No	64 (63.4%)
IVH Grade*	
1	14 (13.9%)
2	36 (35.6%)
3	36 (35.6%)
4	8 (8%)
IVH Onset (days from birth)	
Median (IQR)	3 (5)
Serial Lumbar Puncture	
Yes	23 (22.7%)
No	76 (75.2%)
Ventriculoperitoneal (VP) Shunt	
Yes	37 (36.6%)
No	62 (61.4%)
Associated Symptoms	
Seizures	6 (6%)
Respiratory Distress Syndrome (RDS)	24 (23.7%)
CVS	4 (3.9%)
CNS	3 (2.9%)
Others	1 (1%)
Maternal Characteristics	
Maternal Age (Years)	
Median (IQR)	32 (7)
Gravida	
Primigravida	19 (18.8%)
Multigravida	26 (25.7%)
Mode of Delivery	
Normal Vaginal Delivery	46 (45.5%)
Cesarean Section	34 (33.7%)
Outcomes	
Outcomes	
Neurodevelopmental Delay#	34 (33.7%)
Death	17 (16.8%)

Maternal and neonatal characteristics of posthemorrhagic hydrocephalus (PHH) amongst IVH

Among the 59 (58.4%) neonates who developed PHH, it was resolved in 34 (33.6%) and unresolved in 25 (24.8%). We found a high percentage of males with resolved PHH compared to unresolved PHH (59% vs. 28%; p=0.002). There is no significant difference between the median time of IVH onset for patients with resolved PHH (3±5 days from birth) compared to unresolved PHH (2±5 days from birth; p=0.42). The median birth weight of neonates with resolved PHH was not statistically different from unresolved PHH (1691±1200 vs. 1364±1400; p=0.53). Out of the 59 patients who developed PHH, five (8.5%) had grade I IVH, 26 (44.1%) grade II, 25 (42.4%) grade III, and three (5.1%) with grade IV. A higher frequency of grade III IVH is found in neonates with unresolved PHH compared to those with resolved PHH (60% vs. 29%; p=0.001) (Table 3).

**Table 3:** Maternal and neonatal characteristics and outcomes of post-hemorrhagic hydrocephalus (PHH) amongst intraventricular hemorrhage (IVH) Please note all percentages are column percent to compare resolved PHH and unresolved PHH with other variables. *IVH Grade: 1-Bleeding is confined to the germinal matrix; 2-Germinal matrix hemorrhagic and IVH occupies <50% of the lateral ventricle volume; 3- Germinal matrix hemorrhagic and IVH occupies more than 50% of the lateral ventricle volume and is associated with acute ventricular distension; 4-Periventricular hemorrhagic infarction; Hemorrhagic infarction in periventricular white matter ipsilateral to large IVH; # Neurodevelopmental delay includes all patients with neurological impairment like gross and fine motor skills, language, and cognitive skills.

Variables	Resolved PHH N= 34 (%)	Unresolved PHH N =25 (%)	p-value
Intraventricular hemorrhage (IVH)			
Unilateral			0.37
Bilateral	8 (23%)	3 (12%)	
Laterality Unknown	21 (62%)	19 (76%)	
	5 (15%)	3 (12%)	
Neonatal Characteristics			
Birth weight (Grams)			0.53
Median ± IQR	1691±1200	1364±1400	
Gender			0.002
Male	20 (59%)	7 (28%)	
Female	10 (29.4%)	11 (44%)	
Gestational age			0.07
Term	11 (32.3%)	6 (24%)	
Preterm	23 (67.6%)	19 (76%)	
Ultrasound			0.69
Yes	25 (73.5%)	17 (68%)	
No	9 (26.5%)	8 (32%)	
Magnetic Resonance Imaging (MRI)			0.85
Yes	7 (20.6%)	4 (16%)	
No	27 (79.4%)	21 (84%)	
Computed Tomography (CT) Scan			0.81
Yes	11 (32%)	10 (40%)	
No	23 (67.6%)	15 (60%)	
IVH Grade*			0.001
1	3 (9%)	2 (8%)	
2	19 (56%)	7 (28%)	
3	10 (29%)	15 (60%)	
4	2 (6%)	1 (4%)	
IVH Onset (days from birth)			0.42
Median ± IQR	3±5	2±5	
Serial Lumbar Puncture			<0.001
Yes	17 (50%)	4 (16%)	
No	17 (50%)	21 (84%)	
Permanent Ventriculoperitoneal (VP) Shunt			<0.001
Yes	21 (62%)	16 (64%)	
No	13 (38%)	9 (36%)	
Associated Symptoms			0.007
Seizures	2 (6%)	4 (16%)	
RDS	12 (35%)	12 (48%)	
CVS	3 (9%)	1 (4%)	
CNS	0 (0%)	3 (12%)	
Others	0 (0%)	1 (4%)	
Maternal Characteristics			
Maternal Age (Years)			0.96
Median ± IQR	29±9	30±13	
Gravida			0.69
Primigravida	6 (18%)	5 (20%)	
Multigravida	6 (18%)	7 (28%)	
Mode of Delivery			0.016
Normal Vaginal Delivery	10 (29.4%)	13 (52%)	
Cesarean Section	11 (32.3%)	7 (28%)	
Outcomes			
Outcomes			0.0001
Neurodevelopmental Delay#	8 (23%)	12 (48%)	
Death	1 (3%)	9 (36%)	

In our patients, cranial ultrasound was performed in 42 (71.2%), CT head in 21 (35.6%), and MRI brain in 11 (18.6%). There was no statistically significant association between imaging studies and PHH. We found that unresolved PHH has a higher frequency of seizures, respiratory distress syndrome (RDS), and CNS-related symptoms compared to resolved PHH (16% vs. 6%, 48% vs. 35%, 12% vs 0%; p=0.007), respectively. Furthermore, patients with resolved PHH have higher utilization of serial LP compared to unresolved PHH (50% vs. 16%; p<0.001) (Table 3).

Considering the maternal characteristics evaluated in our study; there was no significant difference between median maternal age for neonates with resolved PHH and unresolved PHH (29±9 vs. 30±13; p=0.96). There was a high frequency of normal vaginal delivery in neonates with unresolved PHH compared to resolved PHH (52% vs. 29.4%; p=0.016). Eleven out of 59(18.6%) of the mothers were primigravida and 13/59 (22%) were multigravida (Table 3).

Univariate analysis of outcomes

We found that neonates with resolved PHH have a lower prevalence of neurodevelopmental delay (NDD) (23% vs. 48%; p=0.0001) and death compared to unresolved PHH (3% vs. 36%; p=0.0001), respectively (Table 3).

Regression analysis

In our adjusted multivariable logistic regression model, we found that neonates with resolved PHH have lower odds of having neurodevelopmental delay (OR:0.15, 95%CI:0.03-0.74; p=0.02) and death (OR:0.9;95%CI:0.01-0.99; p=0.049) compared to unresolved PHH. c-values for the models of neurodevelopmental delay and death were 0.81 and 0.80, respectively, which is >0.7, indicating a good model fit (Table 4).

**Table 4:** Regression analysis of the outcomes of resolved-post hemorrhagic hydrocephalus (PHH) amongst neonates with intraventricular hemorrhage (IVH).

	Model 1 Neurodevelopmental delay					Model 2 Death			
	Odds Ratio (OR)	Confidence Interval (CI)			p-value	Odds Ratio (OR)	Confidence Interval (CI)		p-value
		LL	UL				LL	UL	
Unresolved PHH	Reference								
Resolved PHH	0.15	0.03	0.74		0.02	0.9	0.01	0.99	0.049
Gestational age									
Term	Reference								
Preterm	0.25	0.03	2.02		0.192	0.67	0.061	7.16	0.738
Gender									
Female	Reference								
Male	1.16	0.33	4.04		0.812	0.82	0.19	3.35	0.777
IVH									
Unilateral	Reference								
Bilateral	0.56	0.11		2.75	0.476	1.42	0.17	11.9	0.748
IVH onset	1.02	0.87		1.91	0.839	0.9	0.731	1.11	0.335

## Discussion

The immaturity of the neonatal central nervous system (CNS) results in its vulnerability to injury, particularly when associated with prematurity. The extent of CNS injury can range from IVH (bleeding into the germinal matrix), intraparenchymal hemorrhage (bleeding within the substance of the brain), and white matter injury, including periventricular leukomalacia. The risk of these injuries decreases with increased gestational age, suggesting fragility and immaturity of the premature neonatal brain [1]. Despite the dramatically decreased prevalence of IVH in both term and preterm neonates over the last three to four decades, neonates with severe forms of IVH (grades III and IV) remain at higher risk of developing neurocognitive problems [86-88]. It has been estimated that 60% of premature infants with grade III and IV IVH will incur neurocognitive problems such as developmental delay, cerebral palsy, learning, and intellectual disabilities [2].

In our systematic review, we noted that 59/101 (58.4%) cases with IVH developed PHH. Prematurity was associated with a higher percentage of developing PHH compared to term gestation (17/59, 28.8%). This discrepancy can be attributed to increased maturity of the brain in term neonates and attenuation of underlying risk factors associated with prematurity [3]. Of the 59 patients, resolution of PHH was noted in 34 neonates (57.6%), whereas 25 (42.4%) had unresolved PHH. Furthermore, we found that neonates with resolved PHH have lower odds of developing NDD and death compared to unresolved PHH. This was consistent with findings described by de Vries et al., who noted that neonates with IVH without ventricular dilation had a decreased risk of neurodevelopmental disabilities when compared to those with ventricular dilation, PHH, or IPH [89]. Szpecth et al. in a retrospective analysis identified that approximately 60% of premature infants with grade III and IV IVH with PHH incur neurocognitive problems [2]. We believe that the lower incidence of developing NDD and death in neonates with resolved PHH may be possible as they had a less severe disease when compared to those with unresolved PHH. Secondly, patients with resolved PHH had higher utilization of treatment procedures.

Severe IVH predisposes to ventricular dilation and PHH warranting intermittent spinal or ventricular taps and possible permanent VP shunt placement [86]. There is no general agreement regarding the best treatment option for PHH, and previous literature suggests that there are no standard guidelines that determine when to implant a VP shunt [4]. In our study sample, neonates with resolved PHH had higher utilization of serial LP compared to unresolved PHH (50% vs. 16%, p<0.001), and there was a higher percentage of VP shunt placement in unresolved PHH (62% vs. 64%, p<0.001). The findings reported in our study were similar to those reported by de Vries and Groenendaal et al. and Hong Shen-Lee et al. [5,89]. Published literature recommends that the utilization of these procedures be reserved for symptomatic neonates and long-term neurodevelopmental outcomes of neonates with PHH and permanent shunt have not been systematically determined [4].

Strength and limitations of the study

The major strength of our study is that it analyzed only individual case reports and precisely evaluated the outcomes, including neurodevelopmental delay and death in neonates with PHH. However, our study has some limitations. First, the sample size is small because of the rarity of IVH and PHH in neonates and the strict exclusion criteria, excluding other prospective studies that did not have individual patient data available. Although this was done to avoid duplicate patients and maintain the quality of the article, it further reduced the number of patients in the analysis. Second, there was insufficient data on treatment utilized for PHH in neonates with IVH and the treatment options represent the preferences of the physicians, so we cannot evaluate the role of independent intervention methods in resolving PHH. Nevertheless, given the limited availability of accurate information on this disease, this study shows a relatively large number of patients. Future studies should emphasize the role of early resolution of PHH, the cut-off time to wait before choosing a specific intervention, and the type of intervention to resolve PHH amongst neonates with IVH.

## Conclusions

Our study results show that neonates with resolved PHH have a significantly lower risk of neurodevelopmental delay and mortality. Furthermore, neonates with unresolved PHH have a higher prevalence of seizures, respiratory distress syndrome (RDS), and CNS-related symptoms compared to resolved PHH. In conclusion, our findings highlight the importance of periodic reviews to evaluate the efficacy and safety of neuroprotective strategies designed to mitigate the complications associated with IVH in neonates.
